# Intensifying Effect of Instant Controlled Pressure Drop (DIC) Pre-Treatment on Hesperidin Recovery from Orange Byproducts: In Vitro Antioxidant and Antidiabetic Activities of the Extracts

**DOI:** 10.3390/molecules28041858

**Published:** 2023-02-16

**Authors:** Mariem Ben Abdallah, Morad Chadni, Nouha M’hiri, Fanny Brunissen, Nesrine Rokbeni, Karim Allaf, Colette Besombes, Irina Ioannou, Nourhene Boudhrioua

**Affiliations:** 1 Laboratoire de Physiopathologie, Alimentation et Biomolécules, LR17ES03, Institut Supérieur de Biotechnologie de Sidi Thabet, Université de la Manouba, Ariana 2020, Tunisia; 2 URD Agro-Biotechnologies Industrielles (ABI), CEBB, AgroParisTech, 51110 Pomacle, France; 3 Laboratoire des Sciences de l’Ingénieur pour l’Environnement (LaSIE), UMR-CNRS-7356, Faculté des Sciences et Technologies, La Rochelle Université, CEDEX 1, 17042 La Rochelle, France

**Keywords:** orange byproducts, hesperidin, antioxidant activity, antidiabetic activity, conventional solvent extraction, accelerated solvent extraction, ultrasound-assisted extraction, instant controlled pressure drop

## Abstract

The orange byproduct is a widely accessible and valuable source of functional phenolic compounds, particularly hesperidin. Hesperidin extraction remains a challenging phase in its valorization chain due to its low solubility and limited extractability in solvents. This work aims to examine the effect of conventional solvent extraction (CSE) compared to emerging and innovative extraction methods: accelerated solvent extraction (ASE) and ultrasound-assisted extraction (UAE) when applied with or without a pretreatment process of instant controlled pressure drop (DIC) to intensify extraction, antioxidant, and antidiabetic activities. The total phenols, flavonoids, hesperidin contents, radical scavenging activities, iron chelating activity, and in vitro α-amylase inhibition of the extracts were determined for CSE (80%, 70 °C), UAE (ethanol 80%, 70 °C, 200 W), and ASE (ethanol 60%, 100 °C, 100 bars) with or without DIC pretreatment (pressure = 0.4 MPa, total thermal time = 30 s). The hesperidin amounts obtained were 0.771 ± 0.008 g/100 g DM, 0.823 ± 0.054 g/100 g DM, and 1.368 ± 0.058 g/100 g DM, for CSE, UAE, and ASE, respectively. DIC pretreatment of orange byproducts increased hesperidin recovery by 67%, 25.6%, and 141% for DIC-CSE, DIC-UAE, and DIC-ASE, respectively. The DPPH and ABTS radical scavenging and iron chelating activities of extracts were also significantly enhanced, and the in vitro antidiabetic activity of extracts was preserved.

## 1. Introduction

Citrus is one of the most prominent fruit crops, with a world production of about 158 million tons in 2021. Orange is one of the main varieties grown commercially, producing about 75.6 million tons in 2021. It is also the fourth most consumed fruit in the world [1]. Orange byproduct (peels, seeds, and pulp) represents more than 50% of the fruit weight [2]. Because of its high moisture content (≥75%), the released by-product is conducive to uncontrolled microbial fermentation, causing environmental problems [3,4]. The citrus byproduct is an inexpensive source of natural biomass that is widely accessible and particularly rich in phenolic content, which can vary from 0.70 to 19.6 g/100 g of dry matter according to the extraction method and the operating conditions [3]. Due to their antioxidant properties and capacity to scavenge free radicals, phenolic compounds, particularly flavonoids, have attracted great interest and shown beneficial effects on human health [5,6,7,8,9,10,11,12,13]. Phenolic compounds demonstrated anti-diabetic and hypolipidemic effects by lowering blood glucose and lipid levels. The potential antidiabetic property of phenolic compounds could be attributed to the decrease in gastrointestinal glucose absorption, the inhibition of carbohydrate digestion, stimulation of insulin secretion, modification of liver glucose release, and activation of insulin receptors and glucose uptake in tissues sensitive to insulin [9]. The majority of phenols block amylase and glycosidase activity, which prevents the intestine from absorbing glucose. The interaction between the antioxidative and anti-diabetic effects of phenols remains unknown. Both activities seem independent, but the antioxidative properties of phenols indirectly prevent type 2 diabetes in its early stages by controlling glucose absorption and activating the nuclear receptor transcription factor peroxisome proliferator-activated receptor, PPARα which modulates lipid metabolism and obesity [9].

Synthetic antioxidants present strong constraints because of their limited solubility and moderate antioxidant activity. Therefore, there has been great interest in using natural antioxidants, which have many applications in the food and pharmaceutical industries. One of the primary functional compounds of orange byproducts is hesperidin: a glycosylated flavanone, which is the most prevalent and present in high concentrations of orange peels [2,3]. Hesperidin has several uses in pharmaceutical, cosmetic, and food sectors because of its numerous biological properties such as antioxidant, anti-inflammatory, anti-hypercholesterolemic, anti-hypertensive, hypoglycemic, anticancer, antimicrobial, and antiallergenic properties [14,15,16,17]. In a recent study [17], it was reported when using molecular docking analysis that hesperidin can prevent the SARS-CoV-2 virus from binding to the ACE2 enzyme of the host cell, inhibiting virus replication, and countering the proinflammatory over the reaction of the immune system. The eco-friendly extraction of this bioactive compound from citrus byproducts is challenging to implement. Indeed, despite its high thermal stability (up to 160 °C), it is characterized by low solubility and extractability in water [11,18,19]. Numerous researchers have investigated conventional solvent extraction conditions or more efficient and automated techniques that are more environmentally friendly, requiring less energy and/or solvent while delivering higher yields [2,20,21,22]. Among these methods are microwave-assisted extraction (MAE) [23,24,25,26], enzymatic-assisted-extraction [27], ultrasound-assisted extraction (UAE) [28,29,30,31,32], pressurized liquid extraction (PLE) [33], supercritical fluid extraction (SFE) [25,26], and pressure and electro-based technologies such as the pulsed electric field (PEF) [34], infrared heating coupled to UAE [35], and accelerated solvent extraction (ASE) [36,37]. Ultrasound technology is always used to improve the efficiency of conventional solvent extraction. Ultrasound waves interact with the product and improve biomolecule extractability through a cavitation effect that disrupts the plant cell walls and enhances mass transport. Controlled operating parameters (such as sonication power, time, and ultrasonic wave distribution) allow for the high reproducibility and purity of the final product [32]. ASE allows the extraction of bioactive in a closed and inert environment at pressures varying from 3 to 20 MPa and temperatures ranging from 25 to 200 °C [37]. The desorption and solubility of molecules in solvents are generally increased at temperatures above 40 °C, which promotes the dissolution of phenolic compounds. The pressure promotes contact between the extraction solvent and the product. ASE is receiving more interest from scientists. Indeed, inert atmospheres at high temperatures and pressures allow for a decrease in the organic solvent and oxidation reactions [36]. While phenol and hesperidin recoveries increased in general for severe extraction conditions, antioxidant activity can decrease significantly [25,26]. Thus, the assessment of the target biological activity should be considered along with the increased recovery of the target compound.

The instant controlled pressure-drop technology, or DIC technology, is a thermo-mechanical process that was suggested as an emerging and innovative drying solution to improve the texture, color, odor, techno-functional properties, and microbial safety of dried plants but also to produce notable enhancements over conventional drying and the extraction of biomolecules. DIC allows the treatment of heat-sensitive products at high temperatures and pressures for a brief time based on the principle of instantaneous thermodynamics. For a brief period (5 to 60 s), the sample is heated at high saturation pressures (up to 1 MPa) and high temperatures (up to 180 °C), after which there is an abrupt pressure drop to the vacuum (3–5 kPa, time = 20–200 ms) [38]. DIC-treated plant products generally have better sensory and functional properties based on a significant improvement in mass transfer phenomena. Thus, DIC is also used as a pretreatment to assist and intensify the solvent extraction of phenolic compounds. It consumes less energy and leads to products and extracts that are safe and of improved quality. Indeed, DIC-pretreated fruits and vegetables dry more quickly and develop a porous structure, enhancing the extraction of vegetable oils, essential oils, and antioxidants [38,39,40,41,42,43,44].

The novelty of this work is the exploration of the effect of conventional solvent extraction (CSE) compared to emerging extraction methods; for instance, accelerated solvent extractors (ASE) and ultrasound-assisted extraction (UAE) can be applied separately or in combination with DIC pretreatment to intensify not only the recovery of total phenols and hesperidin from orange byproducts but also to improve and/or preserve the in vitro biological activity of the extracts. The purpose of this work is to investigate (i) the effect of DIC on the CSE, UAE, and ASE techniques and (ii) the antioxidant and in vitro antidiabetic activities of the corresponding extracts.

## 2. Results

### 2.1. Total Phenols, Flavonoids, and Hesperidin Contents of Extracts

**Figure 1:**
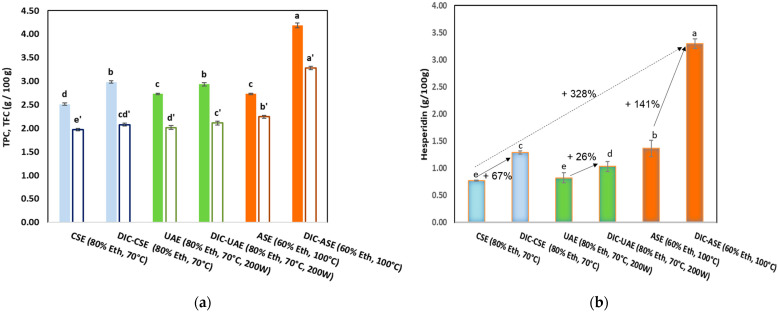
Total phenol contents TPC (g GAE/100 g DM), total flavonoid contents TFC (g QE/100 g DM) (**a**), and hesperidin contents (g/100 g DM) (**b**) of orange byproduct extracts obtained for CSE, UAE, and ASE with or without DIC pretreatment. Values followed by the same letter are not statistically significant according to Tukey’s multiple range test at a significance level of *p* < 0.05.

Figure 1 shows the total phenol contents TPC (g GAE/100 g DM), total flavonoid contents TFC (g QE/100 g DM) (a), and hesperidin contents (g/100 g DM) (b) obtained for CSE, UAE, and ASE with or without DIC pretreatment of the orange byproduct.

The total phenol, flavonoid, and hesperidin contents of the CSE extract were 2.51 ± 0.03 g GAE/100 g DM, 1.97 ± 0.02 g QE/100 g, and 0.770 ± 0.01 g/100 g DM, respectively. The comparison of phenolic contents in the CSE extract to those of UAE and ASE extracts without the DIC pretreatment of the sample showed that TPC, TFC, and hesperidin content significantly increased for UAE and ASE and reached the maximal values for the latter (TPC = 2.73 ± 0.02 g GAE/100 g DM, TFC = 2.24 ± 0.03 g QE/100 g DM, hesperidin content = 1.37 ± 0.01 g/100 g DM). For the three extraction methods (DIC-CSE, DIC-UAE, and DIC-ASE), DIC pretreatment significantly increased the total phenols, total flavonoids, and hesperidin content. Hesperidin content increased by 67% for DIC-CSE, 26% for DIC-UAE, and 141 % for DIC-ASE. When compared to the CSE extraction, which yields the lowest content of 0.770 ± 0.01 g/100 g DM, the DIC-ASE method yielded the highest yield of hesperidin (3.29 ± 0.09 g/100 g DM, i.e., +328%).

### 2.2. Antioxidant and Antidiabetic Activities of Extracts

#### 2.2.1. DPPH, ABTS Radical Scavenging Activities and Iron Chelating Activity

**Figure 2:**
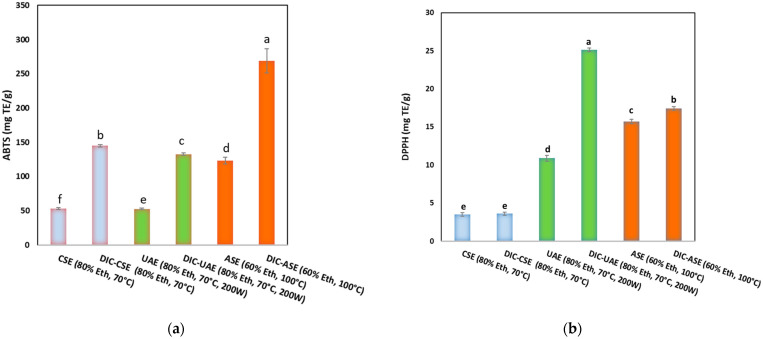
ABTS-RSA (mg TE/g DM) (**a**), DPPH-RSA (mg TE/g DM) (**b**) of orange byproduct extracts obtained for CSE, UAE, and ASE with or without DIC pretreatment. Values followed by the same letter are not statistically significant according to Tukey’s multiple range test at a significance level of *p* < 0.05.

**Figure 3:**
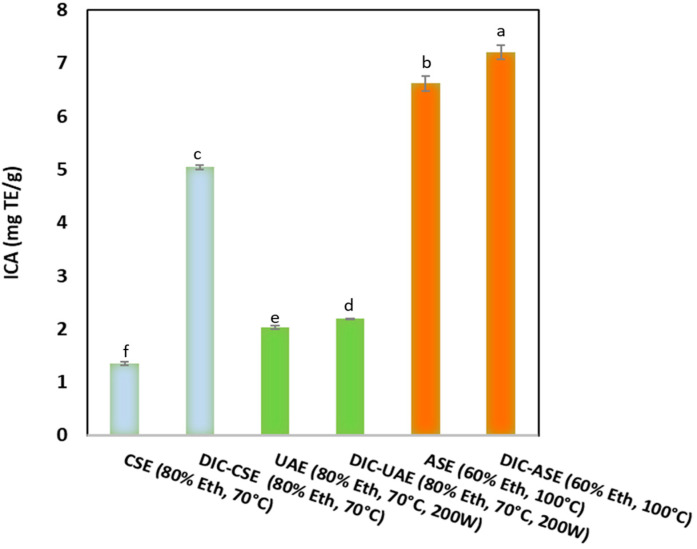
Iron chelating activity (mg EDTA/g DM) of orange byproduct extracts obtained for CSE, UAE, and ASE with or without DIC pretreatment. Values followed by the same letter are not statistically significant according to Tukey’s multiple range test at a significance level of *p* < 0.05.

Figure 2 shows ABTS-RSA (mg TE/g DM) (a), DPPH-RSA (mg TE/g DM) (b), and Figure 3 presents the iron chelating activity (mg EDTA/g DM) of orange byproduct extracts obtained for CSE, UAE, and ASE with or without DIC pretreatment. The antioxidant activities expressed as ABTS, DPPH radical scavenging activities, and iron chelating activity (ICA) showed that orange ethanolic extract was endowed with interesting radical scavenging activity varying from 52.50 ± 1.0 to 269 ± 17.0 mg TE/g DM for the ABTS assay and from 3.5 ± 0.2 to 25.1 ± 0.20 mg TE/g DM for the DPPH assay. In contrast to these high radical scavenging activities, the iron-chelating activities of the orange extracts were low (from 1.35 ± 0.047 to 20 ± 0.10 mg TE/g DM). Whatever the assay used, the lowest antioxidant activity of the orange extract was noticed for CSE. Antioxidant activities were preserved constantly or increased with UAE and ASE. The DIC pretreatment coupled with CSE, UAE, and ASE allowed for a significant improvement in the radical scavenging activities of the extracts and their iron chelating activity compared to the corresponding untreated DIC samples. Indeed, for the ABTS assay, the radical scavenging activity increased by +174% (from 52.9 ± 0.15 to 145 ± 0.018 mg TE/g DM) for the DIC-CSE method, +152% (from 52.5 ± 0.9 to 132.66 ± 0.2 mg TE/g DM) for the DIC-UAE method, and by 118% (from 122.9 ± 0.5 to 268.8 ± 0.1 to mg TE/g) for the DIC-ASE method. A similar trend was observed for DPPH and ICA assays.

#### 2.2.2. In Vitro Antidiabetic Activity

**Figure 4:**
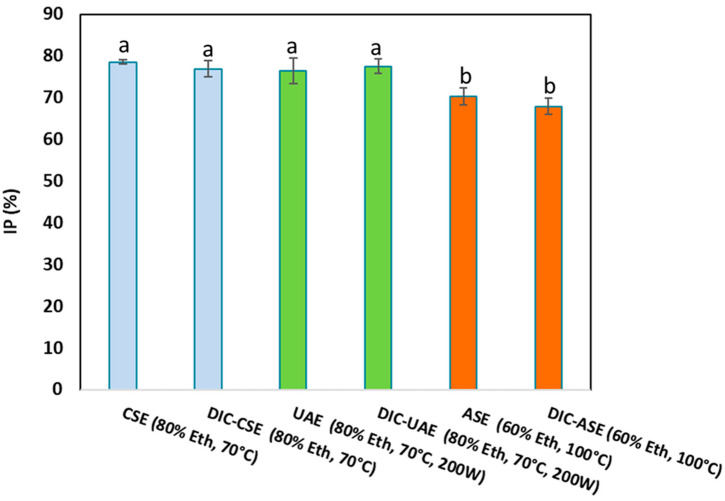
In vitro antidiabetic activity using α-amylase inhibition assay and IP (%) of orange byproduct extracts obtained for CSE, UAE and ASE with or without DIC pretreatment. Values followed by the same letter are not statistically significant according to Tukey’s multiple range test at a significance level of *p* < 0.05.

Figure 4 depicts in vitro antidiabetic activities using the α-amylase inhibition assay for CSE, UAE, and ASE with or without the DIC pretreatment of orange by-product extracts. The α-amylase enzyme was strongly inhibited by the ethanolic extracts of orange byproducts. The percentage of -amylase inhibition ranged from 68 ± 3 to 78 ± 2.5% regardless of the extraction method. ASE and DIC-ASE inhibited α-amylase the least (70 ± 2% for ASE and 68 ± 3% for DIC-ASE). In contrast, no significant differences in -amylase inhibition were observed for CSE, UAE, DIC-CSE, and DIC-UAE.

Consequently, the DIC pretreatment allowed the preservation of the antidiabetic activity of orange byproduct extracts for the different extraction methods (CSE, UAE, and ASE). ASE did not induce any improvement of the in vitro antidiabetic activity, but a slight decrease ranging from 9 to 12% was observed compared to the other extraction methods.

### 2.3. Principal Components Analysis

**Figure 5:**
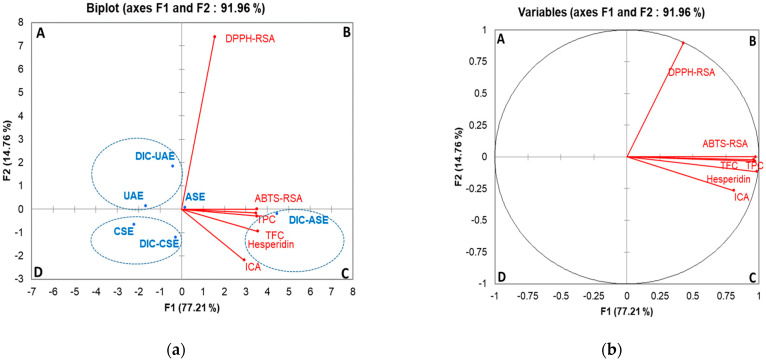
PCA biplot of different extraction methods and measured variables; observations circled in blue indicate distinguished groups (**a**). Biplots of the measured variables: antioxidant activities, and phenol, flavonoid, and hesperidin contents of orange-by products (**b**). A–D are the quadrants of the PCA biplot. CSE-conventional solvent extraction, UAE-ultrasound-assisted extraction, ASE-accelerated solvent extraction, DIC-instant controlled pressure drop.

Figure 5 shows the PCA biplot of the measurements corresponding to the different extraction methods and measured parameters showing significant differences between extracts. The biplot reveals that PCA described 91.96% of data variation through the first two components. Respectively, PC1 explains 77.21% of the variance, and PC2 accounts for an additional 14.76% of the variance (Figure 5a,b). The first dimension is represented positively by ABST-RSA (0.951), ICA (0.656), hesperidin content (0.970), TPC (0.92), and TFC (0.946), whereas the second dimension is represented by DPPH-RSA. Three significant groups of extraction methods were distinguished: extracts of UAE and DIC-UAE form the first group (quadrant A), the extract of CSE and DIC-CSE represent the second group (quadrant D), and DIC-ASE forms another group with a positive correlation of TPC, TFC, hesperidin content, and ABTS-RSA. Table 1 shows the correlation matrix between the measured variables. It is worth noting that all measured parameters showed a strong positive correlation (0.658 ≤ R^2^ ≤ 0.991) with ABTS-RSA. In contrast, DPPH-RSA showed a non-significant correlation with ABTS-RSA and phenols or the hesperidin content.

## 3. Discussion

The total amounts of total phenols, flavonoids, and hesperidin of the orange byproducts obtained for CSE (70 °C, 80% ethanol/water) were in the range of the reported values for Maltease orange peel [25,26]. Both the DPPH-RSA and the ABTS-RSA assays are complementary for determining radical scavenging activity. However, the ABTS assay seems most suitable and more appropriate for selecting the radical scavenging activity of the citrus extract. Indeed, the DPPH assay is reported to be less selective for citrus flavonoids because it does not react with flavonoids that do not contain OH groups in the B-ring as well as with aromatic acids [10,45]. PCA (Table 1) confirmed this observation by showing weak correlations between DPPH-RSA values and TPC, TFC, and hesperidin contents.

The TPC, TFC, and hesperidin recovery show a slight increase with UAE and a higher increase with ASE compared with CSE. These trends agree with the literature [25,26,35,46]. Indeed, the applied ultrasound power, in conjunction with the effect of the temperature, destroys the cell walls of the plant matrix, allowing its content to escape into the solvent. The ultrasonic waves allow the cell walls to collapse through the phenomenon of cavitation, which increases the interaction between the solvent and the phenols. The cavitation bubble generated in the plant material surface induces a microjet directed toward the plant matrix during a compression cycle. This phenomenon seems responsible for the further release of the cellular content into the surrounding solvent [4,26]. The antioxidant activity of the extract was reported to be preserved by using moderate ultrasonic power (≤200 W). Whereas higher ultrasonic powers could induce the formation of free radicals in the liquid medium and accentuate the sonochemical reactions and polymerization/depolymerization reactions, this causes the oxidation of bioactive compounds [24,26]. The ASE gave the highest TPC, TFC, and hesperidin yields and improved antioxidant activities when compared to the UAE and CSE without DIC pretreatment. This can be explained by the high applied temperature (100 °C versus 70 °C for CSE and UAE) and the involved pressure, which is 100 times greater than the atmospheric pressure. A few publications are reporting on the use of the ASE method for phenolic extraction from agri-food byproducts [35,36]. It was also reported that ASE included the better diffusion of the solvent into the sample due to cell-wall disruption upon high pressure and high temperature, inducing a reduced viscosity of the solvent and resulting in better solubility and transport of phenols into the solvent. Indeed, during ASE, the air gaps in fruit tissues are partially filled with liquid. When the pressure is subsequently released, the occluded air in the pores exits, causing plant cell membrane damage. ASE can also cause the deprotonation of charged groups and the disruption of salt bridges and hydrophobic bonds. This makes the cellular membranes less and less selective, thereby rendering the compounds more accessible to extraction up to equilibrium. According to our results, it can be concluded that the yield improvements of TPC, TFC, and hesperidin were associated with an enhancement of the antioxidant activities. A few authors previously conducted ASE for the phenolic recovery of food and agri-byproducts. According to the available literature, ASE can reduce oxidative reactions when used under controlled pressure, temperature, and time conditions [2,35,46].

α-Amylase is an endo-enzyme that catalyzes the hydrolysis of osidic bonds (α-1, 4), and glucose, when absorbed by the gut, is transferred in the portal circulation. Amylase inhibitors in the digestive tract delay the digestion of the polysaccharides, which results in a slower rate of glucose absorption and, as a result, a decrease in blood glucose levels. Flavonoids and other phenolic substances are reported to be potent amylase inhibitors [9,12,13]. Except for the ASE method, all of the extraction methods investigated in this study allowed for the preservation of -amylase inhibition. Indeed, despite improvements in phenol extractability and antioxidant activities (DPPH assay, ABTS assay, and iron chelating activity), ASE and DIC-ASE showed a slight decrease in -amylase inhibition ranging from 9 to 12% when compared to the other extraction methods. The reduction in α-amylase inhibition could be attributed to the orange by-product’s prolonged (30-min) exposure to harsh ASE operating conditions (100 °C and 10 MPa). Shorter extraction times may be favorable for the preservation and/or improvement of this activity. Furthermore, the last observation should be thoroughly investigated using additional in vitro antidiabetic assays, such as α-glucosidase inhibition in vitro.

The solid–solvent interactions are further intensified through DIC pretreatment applied before CSE, UAE, and ASE. Indeed, it is reported that DIC pretreatment induces a higher porosity of the solid matrix, easily removing the internal moisture and increasing the internal solvent transfer and phenols diffusivity [20,39]. This study proved the significant enhancement of TPC, TFC, and hesperidin contents and the antioxidant activities of the DIC pretreated orange byproducts, and the in vitro antidiabetic activity was preserved. It could be explained by the fact that the samples’ exposure to DIC treatment at a saturated steam pressure of 0.4 MPa, implying a high temperature of 143 °C, resulted in a brief processing time (30 s) and was sufficient to implement a texturizing effect by developing a high matrix porosity and protecting the target phenolic compound, and particularly hesperidin.

Indeed, in these conditions, oxygen pressure is ~20 folds lower than that in the ambient atmosphere. Further investigations will be completed to define an effective separation and purification of hesperidin from different extracts (CSE, ASE, DIC-CSE, and DIC-ASE) while exploring in vivo antioxidant and antidiabetic activities. It can be concluded that ASE and DIC treatments are promising methods that could be implemented in the plant byproduct valorization chain to intensify the biomolecule extraction and/or preserve their biological activities.

## 4. Materials and Methods

### 4.1. Plant Material and Sample Preparation

Orange byproducts (*Citrus sinensis* L. *Osbeck*, from the Maltease cultivar) were composed of 80% peel and 20% pulp. The orange by-products had an initial moisture content of 75 ± 1.50 g/100 g wet basis.

### 4.2. Oven Drying of Orange Byproducts

The orange byproducts were dried for 72 h in an oven at 60 °C until reaching a moisture content of 7.50 ± 0.30 g/100 g wet basis. It was finely ground using a blender (Moulinex@, Rosny-sous-Bois, France) and vacuum packed in plastic bags at −20 °C until it was used for conventional solvent extraction (CSE), ultrasound-assisted extraction (UAE), and accelerated solvent-extraction (ASE).

### 4.3. Pretreatment by DIC Dehydration of Orange Byproducts

For the instant controlled pressure-drop DIC process, the orange byproducts were pre-dried in an oven for 20 h at 60 °C until they reached a moisture content of ~16.0 ± 0.50 g/100 g wet basis. Partial drying was required to allow the orange byproduct to be adequate for the DIC treatment [39,41]. The dried byproduct was ground using a blender (Moulinex@, France) and vacuum-packed in plastic bags at −20 °C. DIC equipment is composed of three main elements: a high-pressure/high-temperature processing vessel where the material to be treated is placed; a vacuum system of a large volume tank and water-ring pump; and an instant opening pneumatic valve that assures an abrupt connection between the vacuum tank and the processing vessel [41]. Pre-dried orange byproducts (~16.0 g moisture/100 g wet basis) were placed in a DIC processing vessel to follow one cycle of DIC treatment which consisted of applying a saturated dry steam absolute pressure equal to 0.4 MPa and a total thermal time of 30 s. The sample’s temperature was determined to be 143.67 °C. Five replications were performed for the DIC-pretreatment process.

### 4.4. Extraction Methods

**Figure 6:**
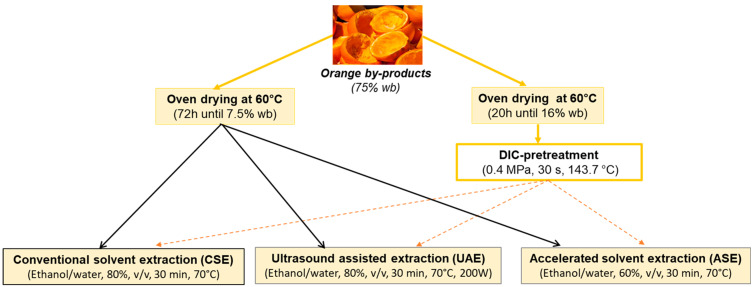
Orange by-products pretreatment and the used extraction methods.

Figure 6 summarizes the experimental methodology and uses extraction methods with or without the DIC pretreatment of orange byproducts. All extraction methods were carried out in triplicate, and the results were given in dry matter (DM).

#### 4.4.1. Conventional Solvent Extraction (CSE)

Five grams of orange byproduct powder were used for extraction with 50 mL of an 80% *v/v* ethanol/water solution. The mixture was shaken at 250 rpm in darkness using a magnetic stirrer for 30 min at 70 °C.

#### 4.4.2. Ultrasound-Assisted Extraction (UAE)

The extraction process was carried out in an agitated glass reactor of 2 L, equipped with an ultrasound probe UP400St (400 W, 24 kHz) and an ultrasound generator (Hielscher, Germany). The extraction temperature was maintained constantly by a circulation of glycerol in an outer jacket connected to a cryothermostat (Lauda, Teltow, Germany). A total of 60 g of orange byproducts were used for the extraction with 600 mL of ethanol/water, 80%, *v*/*v*. (*m*/*v* = 1/10). The agitation speed was fixed at 250 rpm. The extraction was carried out at 70 °C for 30 min with ultrasound power in a continuous mode at 200 W.

#### 4.4.3. Accelerated Solvent Extraction (ASE)

Five grams of orange byproduct powder were extracted using an accelerated solvent extractor (Dionex ASE 350, Thermo Scientific, Sunnyvale, CA, USA) with 50 mL of 60% ethanol/water, *v*/*v*. The extraction was performed at 100 °C for 30 min at 100 bars.

#### 4.4.4. Intensifying Extraction Using DIC Pretreatment

In order to intensify the extraction of the phenolic content and particularly hesperidin content, the DIC-pretreated orange byproduct was used to perform extraction by conventional solvent extraction (DIC-CSE), ultrasound-assisted extraction (DIC-UAE), and accelerated solvent extraction (DIC-ASE) at the operating conditions mentioned above.

### 4.5. Analytical Methods

#### 4.5.1. Determination of Total Phenol and Flavonoid Contents

The crude extracts provided by each extraction method were cooled at room temperature, centrifuged at 4713× *g* for 10 min, and the supernatant was filtered through Millipore paper (0.22 µm). The total phenol content (TPC) was determined by the Folin–Ciocalteu method. The samples were added to the Folin–Ciocalteu reagent and Na_2_CO_3_ solution before being placed in a 40 °C water bath for 30 min. Spectrophotometric analysis (6300PC, VWR, PA, USA) was carried out at 765 nm. TPC was expressed as grams of gallic acid equivalent (GAE) per 100 g of DM [25].

Total flavonoid content (TFC) was determined by a spectrophotometric method. A total of 0.5 mL of the extract was placed in a 5 mL volumetric flask, and then 2.5 mL of distilled water was added, followed by 0.15 mL of 5% NaNO_2_. After 5 min, a volume of 0.15 mL of 10% AlCl_3_ was added. Another 5 min later, 1 mL of 1M NaOH was added, and the volume was made up with distilled water. The solution was mixed, and absorbance was measured at 510 nm using a spectrophotometer (UV-6300PC, VWR, PA, USA). TFC was expressed as grams of quercetin equivalent per 100 g of DM [25].

#### 4.5.2. Analysis of Hesperidin by UHPLC

The quantitative analyses were performed using a UHPLC analytical system (Thermo Scientific Dionex UltiMate 3000, Wilmington, DC, USA) equipped with a quadratic pump, autosampler, column furnace, and diode array detector. Gradient elution was performed using water (solvent A), acetonitrile (solvent B), and formic acid 0.1% (*v*/*v*) in water (C), on a C18 ThermoScientific™Accucore™aQ (100 × 3 mm with 2.6 μm particle size). The column was maintained at 48 °C and ran at a constant flow rate of 0.8 mL/min. The injection volume was 5 µL. The initial solvent (*v*/*v*) was 45% A, 5% B, and 50% C. Solvent C gradient followed as 50% (0 min), 95% (5 min), and 95% (15 min), while B remained constant at 5%. Chromatograms were acquired at 285 nm and analyzed with Chromeleon software (version 6.8). Hesperidin was identified by comparing its relative retention times with its respective standards. A solution of 100 mg/L of hesperidin was prepared by solubilizing in a mixture of ethanol and DMSO (1:250 *v*/*v*), which was heated in an ultrasonic bath for 10 min to enhance the solubilization. Standards were then prepared by diluting with ethanol, and samples were filtered through Millipore paper (0.22 µm) prior to UHPLC injection.

#### 4.5.3. Determination of Radical Scavenging Activity by DPPH Assay

The radical scavenging activity of orange byproduct extracts was evaluated using the DPPH assay [30]. A 63 µM of 1.1-diphenyl-2-picrylhydrazyl (DPPH) was prepared by diluting 2.5 mg of DPPH with 100 mL of methanol. A total of 400 µL of the sample extract was added to 2.4 mL of DPPH solution and incubated in the dark for 30 min at room temperature. The reduction in the DPPH radical was determined by measuring the absorption at 515 nm. The DPPH assay was calibrated by the linear range of Trolox concentrations (0–50 µg/mL). The DPPH radical scavenging activity (DPPH-RSA) was expressed as the mg Trolox Equivalent (TE/g DM). All samples were investigated in triplicate.

#### 4.5.4. Determination of Radical Scavenging Activity by ABTS Assay

The ABTS-radical scavenging activity (ABTS-RSA) of different extracts was evaluated using the method described by M’hiri et al. [25] with minor modifications. The radical cation of 2,2-azinobis-3-ethylbenzothiozoline-6-sulphonate and ABTS radical cation was produced by reacting 7 mM of ABTS stock solution with 3 mM potassium persulfate (K_2_S_2_O_3_). The mixture was incubated in the dark at room temperature for 12 h. To determine the ABTS-RSA, 50 μL of the diluted extract was mixed with 2mL of ABTS solution and left at room temperature for 30 min. The absorbance was measured at 734 nm. The ABTS assay was calibrated by the linear range of Trolox concentrations (0–250 µg/mL). ABTS-RSA was expressed in mg Trolox equivalents, TE/g DM. All samples were investigated in triplicate.

#### 4.5.5. Iron Chelating Activity 

The iron chelating activity (ICA) of different extracts was estimated by the method described by Oboh and Ademosun [47] with slight modifications. Thus, 500 μL of the extract and 50 μL of FeSO_4_7H_2_O (2 mM) were mixed. Then, 200 μL of ferrozine (5 mM) was added. After an incubation time of about 15 min at 25 °C, the absorbance was measured at 562 nm. The assay was calibrated by the linear range of EDTA chelating agent concentrations (10–90 µg/mL). The ICA values were given in mg EDTA equivalents/g dry matter (mg/g DM). All samples were investigated in triplicate.

#### 4.5.6. In Vitro α-Amylase Inhibition Assay

The chromogenic dinitrosalicylic acid (DNS) method described by Benayad et al. (2021) [12] was used with minor modifications to test the inhibitory activity of orange by-product extracts against α-amylase. Before use, the porcine pancreatic α-amylase enzyme solution was freshly prepared in a sodium phosphate buffer (20 mM, pH = 6.9) at a concentration of 5 units/mL. The inhibitory assay began with the addition of 500 µL of α-amylase to various extracts. In a shaker incubator (Thermo Scientific ^TM^, Illkirch-Graffenstaden, France), samples were pre-incubated for 15 min at 37 °C.

The reaction was initiated by adding 1% starch substrate solution to all the tubes. Then, the mixture was incubated at 37 °C for 30 min in a boiling water bath. The reaction was stopped by adding 1 mL of 3,5-dinitrosalicylic acid (DNS). Then, the mixture was heated for 15 min in a boiling water bath and cooled down in an ice bath. Finally, the reaction mixture was diluted by adding 5 mL of distilled water. The absorbance was measured at 540 nm. The blank sample was prepared following the same steps as above, but the extract was replaced by 500 µL of the sodium phosphate buffer. Acarbose was used as a positive control. The α-amylase inhibitory percentage was determined as follows:

(1) $$IP\;\left(\%\right)=\frac{A_{S}-A_{0}}{A_{S}}\times 100$$

where $A_{S}$ and $A_{0}$ represent the absorbance of the control (blank) and sample extract, respectively.

### 4.6. Statistical Analysis

Statistical analysis was carried out using XLStat software version 2019 (Addinsoft, Paris, France). The comparison of averages of each treatment was based on the analysis of variance (ANOVA) at the significance level of 5%. Values followed by the same letter are not statistically significant according to Tukey’s multiple range test at a significance level of *p* < 0.05. Principal components analysis was also performed on the measured parameters (TPC, TFC, hesperidin content, DPPH-RSA, ABTS-RSA, ICA), showing significant variations with the used extraction methods (CSE, UAE, ASE, DIC-CSE, DIC-UAE, and DIC-ASE).

## 5. Conclusions

The intensifying effect of DIC pretreatment on phenols, flavonoids, and hesperidin extraction from orange byproducts by coupling it with conventional extraction (CSE), ultrasound-assisted extraction (UAE), and accelerated solvent extraction (ASE) was assessed. Orange byproducts pretreated or not by the DIC process (0.4 MPa, 30 s, 143 °C) were used for CSE, UAE, and ASE. Hesperidin amounts followed a degressive evolution: of DIC-ASE > ASE > DIC-CSE > DIC-UAE > UAE > CSE.

DIC-ASE had the highest ABST radical scavenging activity and iron chelating activity, while DIC-UAE had the highest DPPH-radical scavenging activity. Orange extracts inhibited amylase at a high level (≥67%), and this activity was maintained after DIC treatment. When compared to the other extraction methods (CSE, UAE), ASE caused a slight decrease in α-amylase inhibition ranging from 9 to 12%. DIC pre-treatment, combined with CSE, UAE, and ASE, seems to be a promising approach for the enhancement of the extraction yield of biomolecules from plant byproducts with the improvement or preservation of in vitro antioxidant and antidiabetic activities.

## Figures and Tables

**Table 1:** Pearson correlation matrix of TPC, TFC, hesperidin content, and antioxidant activities.

Variables	DPPH-RSA	ABTS-RSA	ICA	Hesperidin	TPC	TFC
DPPH-RSA	1	0.413	0.177	0.306	0.361	0.367
ABTS-RSA	-	1	0.787	0.944	0.945	0.913
ICA	-	-	1	0.776	0.658	0.720
Hesperidin	-	-	-	1	0.970	0.991
TPC	-	-	-	-	1	0.962
TFC	-	-	-	-	-	1

DPPH-RSA: DPPH radical scavenging activity, ABTS-RSA: ABTS radical scavenging activity, ICA: iron chelating activity, Hesperidin: hesperidin content, TPC: total phenols content, TFC: total flavonoids content.

## Data Availability

Data is contained within the article.

## Sample Availability

Samples of the compounds are available from the authors.
